# Ancient horizontal gene transfer and the last common ancestors

**DOI:** 10.1186/s12862-015-0350-0

**Published:** 2015-04-22

**Authors:** Gregory P Fournier, Cheryl P Andam, Johann Peter Gogarten

**Affiliations:** Department of Earth, Atmospheric and Planetary Sciences, Massachusetts Institute of Technology, Cambridge, MA 02139 USA; Department of Epidemiology, School of Public Health, Harvard University, Boston, MA 02115 USA; Department of Molecular and Cell Biology, and the Institute for Systems Genomics, University of Connecticut, 91 North Eagleville Road, Storrs, CT 06269-3125 USA

**Keywords:** Horizontal gene transfer, Aminoacyl-tRNA synthetase, Cenancestor, LUCA, Last Universal Common Ancestor, Hypnolog

## Abstract

**Background:**

The genomic history of prokaryotic organismal lineages is marked by extensive horizontal gene transfer (HGT) between groups of organisms at all taxonomic levels. These HGT events have played an essential role in the origin and distribution of biological innovations. Analyses of ancient gene families show that HGT existed in the distant past, even at the time of the organismal last universal common ancestor (LUCA). Most gene transfers originated in lineages that have since gone extinct. Therefore, one cannot assume that the last common ancestors of each gene were all present in the same cell representing the cellular ancestor of all extant life.

**Results:**

Organisms existing as part of a diverse ecosystem at the time of LUCA likely shared genetic material between lineages. If these other lineages persisted for some time, HGT with the descendants of LUCA could have continued into the bacterial and archaeal lineages. Phylogenetic analyses of aminoacyl-tRNA synthetase protein families support the hypothesis that the molecular common ancestors of the most ancient gene families did not all coincide in space and time. This is most apparent in the evolutionary histories of seryl-tRNA synthetase and threonyl-tRNA synthetase protein families, each containing highly divergent “rare” forms, as well as the sparse phylogenetic distributions of pyrrolysyl-tRNA synthetase, and the bacterial heterodimeric form of glycyl-tRNA synthetase. These topologies and phyletic distributions are consistent with horizontal transfers from ancient, likely extinct branches of the tree of life.

**Conclusions:**

Of all the organisms that may have existed at the time of LUCA, by definition only one lineage is survived by known progeny; however, this lineage retains a genomic record of heterogeneous genetic origins. The evolutionary histories of aminoacyl-tRNA synthetases (aaRS) are especially informative in detecting this signal, as they perform primordial biological functions, have undergone several ancient HGT events, and contain many sites with low substitution rates allowing deep phylogenetic reconstruction. We conclude that some aaRS families contain groups that diverge before LUCA. We propose that these ancient gene variants be described by the term “hypnologs”, reflecting their ancient, reticulate origin from a time in life history that has been all but erased”.

**Electronic supplementary material:**

The online version of this article (doi:10.1186/s12862-015-0350-0) contains supplementary material, which is available to authorized users.

## Background

### Horizontal gene transfer and evolutionary innovation

Horizontal gene transfer (HGT) enables organisms to acquire pre-existing adaptive characters from other organisms, regardless of phylogenetic distance. Thus, instead of genetic traits within lineages always emerging gradually through successive mutations and selection, evolution is accelerated as a parallel process, where inventions made in different lineages can come together in a single cell through HGT. Several cases of transfers that conferred a specific novel adaptation to the recipient lineage have been documented. For example, genes that encode proteins involved in plant cell wall degradation in plant-parasitic nematodes originated from different bacterial sources [1]. In addition to sharing metabolic capabilities between unrelated organisms, HGT also plays an important role in creating new functional roles for existing proteins by assembling new metabolic pathways. Some pathways that changed the face of planet Earth, such as acetoclastic methanogenesis in *Methanosarcina* [2,3] were likely assembled through gene transfer. All enzymes involved in the newly identified methylaspartate cycle for acetyl-CoA assimilation in Halobacteriales were acquired through the horizontal transfer and recombination of different pre-existing genes from different bacterial genomes [4]. These genes were originally involved in various metabolic processes, such as glutamate fermentation and propionate assimilation, leading the authors to call the cycle a “metabolic patchwork”. Gene family expansion often provides the starting material for proteins to adapt to new functions. However, in prokaryotes this expansion rarely occurs as autochthonous within-lineage gene duplication events; rather, new gene family members are acquired through HGT [5,6].

Mobile genetic elements, including transposons, plasmids, bacteriophage and self-splicing molecular parasites, have played a crucial role in facilitating the movement of genetic material between organisms [7,8]. These elements likely already played a similar role in the early stages of life’s evolution [9], and continue to play a role even in multicellular eukaryotes. For example, pervasive transfer of rolling-circle transposons (also called helitrons; [10]) has been reported to occur in many species of insects, plants and vertebrates, primarily through insect viruses as vectors [11]. During transposition, helitrons often capture genes or gene fragments from their host, and can therefore significantly influence the evolution of their host genome, for example through modifications of the transcriptome and in generating new genes [12]. These parasitic entities have been implicated in altering structural, functional and epigenetic variability of their host genome [13], consequently enhancing the evolvability of species and lineages. The persistence of these molecular parasites in the genomes of their hosts may reveal an evolutionary arms race [14], and in some cases, molecular domestication has been reported [15,16].

According to Darwin’s theory [17], natural selection is the differential success in reproduction of individuals in a population. The “fittest” organisms produce more offspring and as a consequence, the traits that produce this fitness become dominant in the population leading to evolution of the population. Dawkins [18] argued that all genes are selfish, yet most cooperate with other genes to build the organisms that carry the genes into future generations. In contrast to the genes, which are selected together via the organismal phenotype, molecular parasites such as transposons, self-splicing introns, and inteins possess their own life cycle [16]. Their selective advantage (i.e., more gene copies in the next generation) does not necessarily correspond to a fitness increase of the host [19]. Thus, such genes can be driven to fixation in the population even if their associated traits are slightly deleterious to the organisms carrying them. These molecular parasites provide examples for nearly neutral pathways to increased complexity, as was suggested for spliceosomal introns and split inteins [16,20]. Transfer of genes that provide adaptation to environmental conditions has been interpreted as altruism on the part of the organism sharing the gene, as the donor lineage does not increase in fitness as a consequence of the transfer. However transfer of genes via passive uptake of genetic material from the environment is not under the control of the donor lineage, and thus does not constitute an act within a community that could be described as altruistic. This may most often be the case for transfers between distantly related groups that do not share physiological mechanisms for gene exchange. Nevertheless, the exchange of genetic information within populations and communities connects selection at the level of the gene, the individual, and the community [21].

Throughout the history of life, HGT has played an important role in gene and genome evolution. Ancient HGT during Hadean/Archaean times is more difficult to study than more recent transfers, although it has been proposed that its role was even more pronounced during earlier times in life’s history [22]. This difficulty is, in part, due to our lack of knowledge about this primordial world – we remain largely ignorant of the lineages that existed then, their genetic makeup, physiological and metabolic capabilities, and the ecological niches available to them. With the vast majority of species that have ever existed on earth now extinct, it also becomes challenging, if not impossible, to identify the donor and/or recipient organisms in extremely ancient HGT events, especially those that occurred during and prior to the time of LUCA. However, the explosion of genome sequencing data, combined with deep paralog phylogenies, can now give us at least a partial snapshot of the genetic composition of some of these extinct lineages. Due to patterns of speciation and extinction across geological timescales, it is expected that many gene acqusitions via HGT, especially those occurring in the distant past, are from lineages that have since gone extinct [23,24]. For the earliest HGT events, deeply branching paralogous gene families are the expected result and evidence of such events. Under the hypothesis of a community of organisms that lived alongside LUCA as independent lineages, these likely shared genetic material with each other through HGT. Therefore, this predicts that some genetic material within extant organismal lineages is not descended from LUCA, but originated in various deep lineages with shared ancestry even further in the past. These patterns of HGT may help explain deep incongruences between gene and species trees at the Domain level.

### The modern genetic code predates LUCA

HGT depends upon the universality of the genetic code. Every known organism uses the same twenty amino acids (the infrequent usage of an additional two, selenocysteine and pyrrolysine, notwithstanding), and with few exceptions these are decoded by tRNA in the same way. Without a universal set of amino acids and decoding system, genes transferred between organisms could not be translated and expressed as proteins, and lineages would be isolated by their own distinct “genetic dialect”. This isolation likely exists today for some lineages that use different codons for some amino acids [25]. Perhaps more commonplace are substantial differences in codon usage and tRNA abundances driven by varying genome nucleotide compositions that may also create a significant hurdle to successful expression and positive selection following transfer [26]. However, these differences in genetic dialect can be overcome with sufficient selective advantage, and lack the qualitative incompatibility that distinct genetic codes impose. It is clear that, in order for HGT to exist before and during the time of LUCA, a universal genetic code must also exist. There is substantial empirical evidence that suggests this is indeed the case, given the near-universality of amino acid biosynthesis and aminoacylation systems, as well as empirical studies of amino acid usage in reconstructed protein sequences mapping to LUCA [27]. At least one essential component of the enforcement of the genetic code, tRNA modification, shows major differences between domains. This has been proposed to indicate a non-universal origin of the code, post-LUCA [28,29]. However, taken together, these observations suggest that a more parsimonious explanation is the early emergence of tRNA modification, with subsequent functional replacements in the branches leading to different domains. Certain chemical similarities between these modifications make this scenario intuitively satisfying, as there is no *a priori* reason for convergent evolution to discover such similar modification substrates. For example, both lysidine and agmatine modifications of tRNA^Ile^ require the attachment of a positively charged amino acid in a similar fashion (lysine or arginine, respectively). Even a universal code must have progressed through evolutionary stages of increasing complexity. The presence of HGT early in the evolution of life before the time of LUCA is also supported by the optimality of the genetic code itself, which likely depended upon extensive HGT to become established [30].

### On the possibility of ancient HGT involving pre-LUCA lineages

The universality of the genetic code in primordial lineages is likely both a product of and precondition for HGT occurring before, during, and after the time of LUCA. As such, some transfers from lineages diverging before LUCA would have been to the ancestors of extant lineages. Due to patterns of extinction and coalescence, these HGT events would manifest themselves as unusually deeply branching divisions within gene trees, leading to rare and unusual protein homologs with much narrower phylogenetic distributions than their sister clades. This scenario requires deeply branching lineages surviving well beyond the time of LUCA, so that transfer could occur to lineages derived from LUCA. Extinction of the donor lineage could then obscure the origin of these rare gene types.

## Results and discussion

### Rare forms of canonical aaRS: Pre-LUCA origins and HGT

aaRS are ancient families of enzymes, present in the organismal LUCA and an integral, fundamental component of the translation machinery, covalently linking amino acids to cognate tRNAs. In this way, aaRS define the interpretation of the triplet anticodons specified in the genetic code, and therefore it has been proposed that their evolution is closely associated with at least some development of the code [31]. However, the process of aminoacylation is thought to have emerged much earlier, with ribozymes acting as catalysts during the RNA world [32]. A period between ribozyme-mediated aminoacylation and the emergence of the cellular LUCA might have relied upon different combinations of tRNAs, amino acids and aaRS as the genetic code was improved. Consequently, the contemporary pool of canonical aaRS might have been significantly different from what was present in primordial times. Today, rare forms of canonical aaRS and other atypical synthetases have a restricted distribution in extant life; their origins might be traced to a deep genealogical record of early, pre-LUCA living systems.

aaRS are divided into two major families, class I and class II, that differ in their aminoacylation mechanisms and in the architecture of their active sites [33]. Their unrelated structures may suggest two independent origins of the aminoacylation mechanism [34]. A single ancestral form gave rise to all the members of each aaRS class, despite differing specificity for amino acids, through several duplication and divergence events [35]. The synthetases of each class can be further divided into three subclasses (designated as Ia, Ib, Ic, IIa, IIb, IIc), whose members are thought to be more closely related to each other than to other aaRS of the same class [36]. The families within each subclass recognize amino acids that are chemically related (*e.g.* hydrophobic, charged, aromatic) which is also likely a product of their common ancestry [31].

### PylRS: ancient origins of non-canonical aaRS

**Figure 1:**
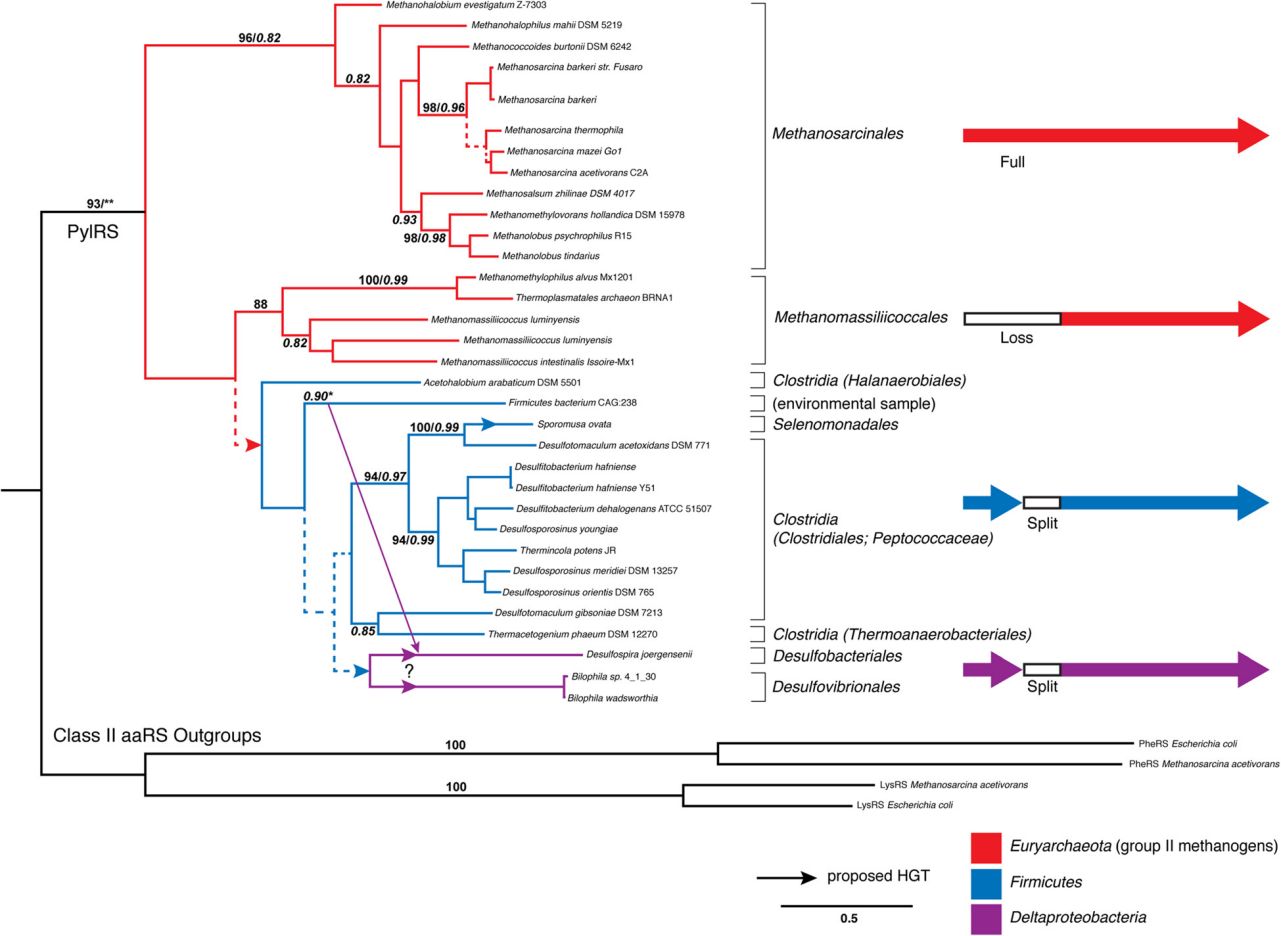
Maximum-likelihood (ML) tree of known PylRS homologs. Branch colors depict the taxonomy of PylRS lineages, with Euryarchaeota (red), Firmicutes (blue) and Deltaproteobacteria (magenta). The different observed protein subunit configurations are depicted on the right, using the same color scheme. Absent protein regions within some groups is represented by whitespace. More narrow taxonomic designations are bracketed and labeled. The tree is rooted by an outgroup consisting of aligned regions from other class II aaRS representatives from different subclasses. Dotted lines represent branches with <50% bootstrap (BS) support; major clades with >80% BS support are labeled with respective BS support values. Italic numbers represent posterior probability (PP) support values from tree reconstruction in Phylobayes3.3 (PB). Significant conflict between the ML and PB trees is shown by an alternative diagonal branching. The alignment contains 37 taxa with alignment length of 475 amino acids. Branch lengths depict substitutions/site. (*)PP support value in the alternative PB topology; (**)PP support was not recovered for the rooting of PylRS using PB, as this node remained unresolved.

Some rare aaRS protein families show an atypical phylogenetic signal suggestive of ancient HGT from extinct lineages. Pyrrolysyl-tRNA synthetase (PylRS) is an enzyme that charges tRNA^Pyl^ with the non-canonical amino acid pyrrolysine (Pyl) [37]. This rare enzyme has a very restricted phylogenetic distribution, suggesting a cryptic origin and a history involving HGT [23,38,39]. In relation to the other aaRS, PylRS is placed as a deep-branching lineage within the subclass IIb, diverging outside other aaRS families. Previous phylogenetic analyses showed that PylRS was found only in members of the archaeal order Methanosarcinales, the firmicute *Desulfitobacterium hafniense* and a Deltaproteobacterial endosymbiont [38,40,41]with a rooting between the archaeal and bacterial groups [23]. The bacterial homologs of this protein are present as two separate genes encoding the N-terminal and C-terminal regions of the protein, respectively. More recent genome sequencing projects have revealed the presence of PylRS within additional genomes, expanding its known phylogenetic distribution and revealing additional clues to its evolutionary history (Figure 1). PylRS has now been identified within additional groups of Firmicutes including *Desulfosporosinus*, *Desulfotoma*, *Sporomusa*, *Thermincola*, *Thermacetogenium*, and *Acetohalobium*. Within Archaea, PylRS has been additionally identified within a newly discovered order of methanogens closely related to Thermoplasmatales, the proposed group Methanomassiliicoccales [42]. Interestingly, PylRS within Methanomassiliicoccales is apparently entirely lacking this N-terminal region, which does not seem to be encoded by a separate gene within their genomes. It has been experimentally shown that tRNA recognition and aminoacylation can occur in the absence of this N-terminal domain *in vitro* [43].

Phylogenetic analysis shows a major division within the PylRS protein family, between Methanosarcinales and a Methanomassiliicoccales/Bacteria clade (Figure 1). Given the close phylogenetic relationship of the two methanogenic clades within the euryarchaeal tree, this suggests vertical inheritance of the Pyl system within group II methanogens, with a subsequent HGT to *Bacteria* along the branch leading to Methanomassiliicoccales. However, Methanomassiliicoccales and Bacteria are very closely related within this phylogeny, and as a likely consequence of this short branch their relationship is not well resolved. In fact, PylRS within Methanomassiliicoccales shares several specific amino acid residues and at least two indels with the deepest branching bacterial sequence, *Acetohalobium arabiticum*. Therefore, it is also possible that Pyl usage in Methanomassiliicoccales is the result of an additional secondary HGT from within Bacteria, although this is a less parsimonious explanation. The distribution of the Pyl system within Clostridia can be explained by gene loss, HGT, or some combination of both, with at least one additional HGT to the ancestor of a subset of Deltaproteobacteria. These results and conclusions are consistent with those from another recently published phylogenetic analysis of PylS [44]. While this analysis did not include aaRS outgroups to root the recovered phylogeny, similar scenarios of HGT were proposed between the archaeal and bacterial gene variants, and Borrel et al. also further suggest that PylS and Pyl utilization originated within methanogenic archaea [44]. The placement of PheRS and LysRS outgroups supports this hypothesis under maximum likelihood tree reconstruction. While the placement of outgroups is uncertain and results in a polytomy under Bayesian models of reconstruction (see Additional file 1), there is only 12-19% posterior probability support for placing the outgroup outside of the methanogens, as would be expected in the case of a bacterial ancestor.

The narrow taxonomic usage of Pyl is accompanied by an extremely narrow functional role, at single catalytic sites in only a handful of proteins required for methylotrophy (mono-, di- and trimethylamine methyltransferases), none of which are homologous to one another [45,46]. The evolution of an entirely distinct genetically-encoded amino acid for such a singular and specialized function is unprecedented in any other known translation systems. Combined with the observed phylogenetic distribution, this suggests an origin outside of the methanogens, in a deep lineage where the Pyl system would have evolved along with a broader biochemical utility for this novel amino acid, leading to its retention. HGT of the Pyl system along with Pyl-dependent enzymes would suffice for the retention of the system in a class II methanogen ancestor, facilitating the evolution of methanogenesis from methylamines. If PylRS was a recent invention within methanogens, one would expect the PylRS protein family to have diverged from another methanogen aaRS protein family, and thus be a derived lineage within known aaRS groups for this clade. In fact, the phylogenetic placement of PylS in relation to other aaRS suggests this donor lineage would branch deeper than LUCA, an ancient, unknown, and possibly extinct lineage. Additional phylogenetic analyses of methylamine methyltransferase protein families and improved environmental sequencing are needed to further evaluate this hypothesis.

The only other sparsely distributed, noncanonical aaRS family is O-phosphoseryl tRNA synthetase (SepRS), which charges tRNA^Cys^ with O-phosphoserine (Sep) to form phosphoseryl-tRNA^Cys^, which is then converted to Cys-tRNA^Cys^ [47]. Found only in methanogens and Archaeoglobales within Euryarchaeota, some of these genomes possess only the SepRS system for tRNA^Cys^ aminoacylation (*e.g. Methanopyrus kandleri*), while some carry both SepRS and CysRS (*e.g. Methanosarcina* species). Phylogenetic analysis reveals that the SepRS protein family roots more deeply than the duplication event that gave rise to the two PheRS subunits before the organismal LUCA [48]. This taxonomic distribution of CysRS suggests a scenario analogous to that proposed for PylS, in which CysRS was inherited vertically from LUCA in most lineages, but displaced in some by SepRS inherited from an ancient HGT event.

Mechanisms of aminoacylation must have evolved very early in the history of life, concurrent with the emergence of tRNA-based template directed translation. Since only one lineage from the primordial biosphere gave rise to the three domains of life (*i.e.* the organismal LUCA), it is likely we have only inherited a subset of the coding diversity that may have existed at this time. Furthermore, due to HGT, the most recent common cellular ancestor and the most recent common ancestors of any particular gene did not necessarily coincide in time and space [49]. Therefore, rare and atypical forms of aaRS, such as SepRS and PylRS, may be remnants from a time when translation machineries were more diverse. In explaining the unusual cases of these aaRSs, this hypothesis predicts that divergent homologs of other canonical aaRS families would also have been transferred into surviving lineages, appearing as deeply branching “rare” forms. We observe that, for several aaRS families, this indeed appears to be the case.

### Atypical canonical aaRS types

**Figure 2:**
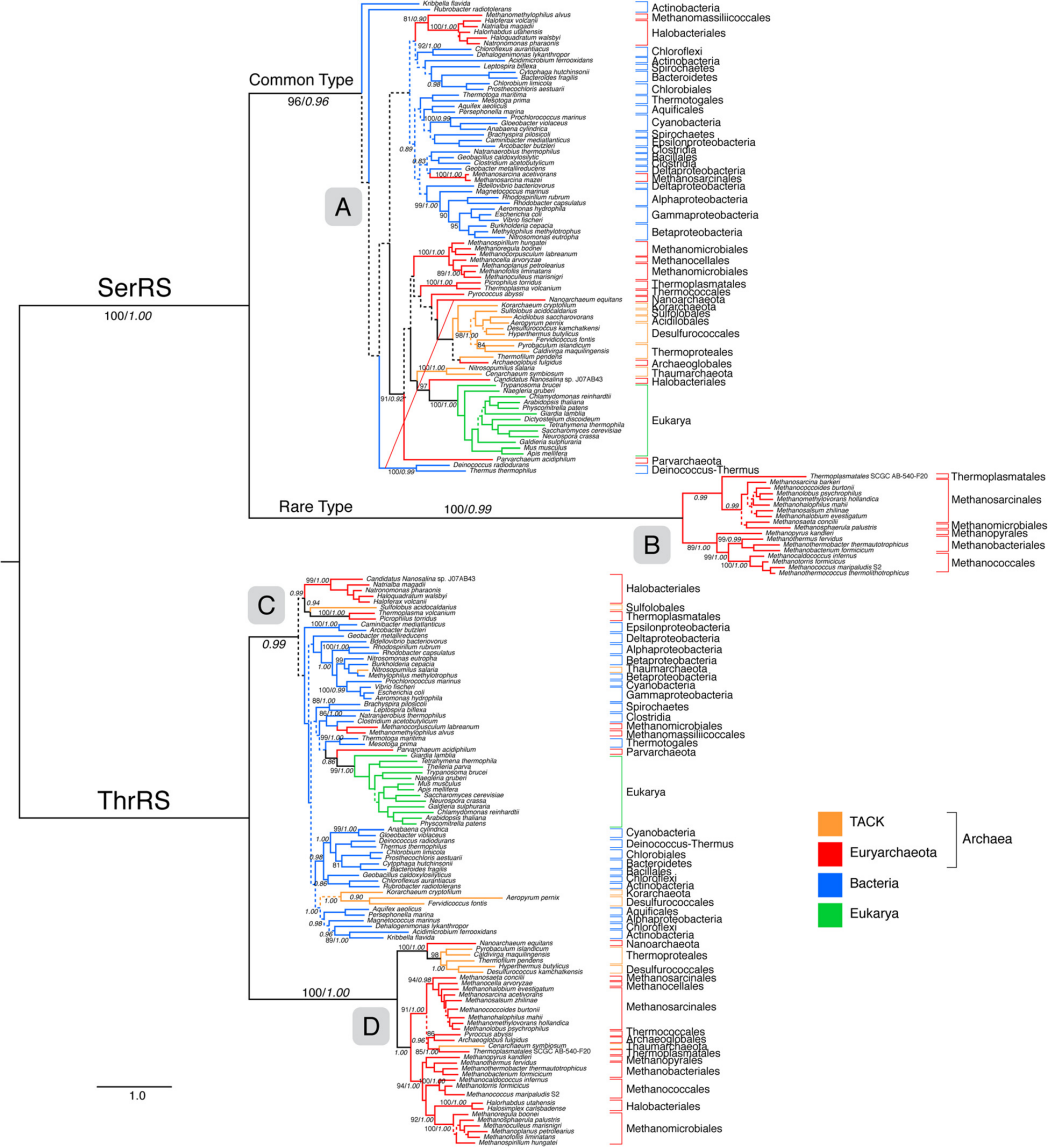
ML tree showing the common and rare types of SerRS and ThrRS. Branch colors depict Euryarchaeota (red), TACK groups (Thaumarchaeota, Crenarchaeota, Korarchaeota) (orange), Bacteria (blue) and Eukarya (green). More narrow taxonomic designations are bracketed and labeled. Ancestral nodes of rare and common aaRS types within the tree are labeled **(A-D)**. Dotted lines represent branches with <50% BS support; major clades with >80% BS support are labeled with respective BS support values. Italic numbers represent posterior probability (PP) support values from tree reconstruction in PB. Significant conflict between the ML and PB trees is shown by an alternative diagonal branching. The full alignment used for the ML tree contains 201 taxa, an alignment length of 1322 amino acids and was reciprocally rooted using each aaRS family paralog. For the PB reconstruction, a decomposed alignment was used, concatenating global and aaRS family-specific well-aligned sites as identified by GUIDANCE for a total alignment length of 453 amino acids (see methods). Branch lengths depict substitutions/site. (*)PP support value in the alternative PB topology.

Within two aaRS families, seryl- (SerRS) and threonyl-tRNA synthetases (ThrRS), an atypical form exists that is distributed in only a subset of archaeal lineages (Figure 2). For each of these aaRSs, the rare and common types form well-supported distinct clades. These atypical enzymes exhibit low sequence and structure similarity to their more common counterparts that are found in a majority of organisms within the three domains of life [50], although they are still clearly homologous and share a common ancestor with other aaRS within their respective families. Despite their differences, the rare and common forms perform identical roles, ligating Ser and Thr to their cognate tRNA molecules.

Both ThrRS and SerRS are members of the Class IIa aaRS. To account for the more narrow distributions of the rare forms of SerRS and ThrRS, we propose that the rare forms of these two enzymes diverged early from the common forms through speciation events occurring before LUCA. Phylogenetic reconstruction based on representatives from several Class II aaRS shows that an ancient divergence gave rise to the common and rare forms of both ThrRS and SerRS (Figure 2). This divergence event appears to have occurred prior to the ancestor nodes for the common forms of SerRS and ThrRS (defined as the common ancestor of bacterial, eukaryal and archaeal homologs). This implies that other unknown or extinct lineages co-existed at the time of the organismal LUCA [49,50], and persisted long enough to transfer genes to early lineages within extant domains, after the diversification following LUCA. In the case of SerRS and ThrRS, the ancestors of some archaeal phyla appear to have been the recipients of these rare aaRS types (Figure 2).

### Phylogenetic distributions within SerRS and ThrRS common and rare types

Within the common and rare types of SerRS and ThrRS are numerous interdomain HGT events that complicate inferring the mapping of the ancestral node of each type to a species tree (Figure 2). The common type of SerRS (A) contains all bacterial groups. Additionally, from within the bacterial clades there are at least 3 HGT events to euryarchaeal lineages, including Halobacteriales, Methanomassiliicoccales, and *Methanosarcina*. While the precise placement of the root of the common type is uncertain due to short internal branch lengths and the large phylogenetic distance to the outgroup, the major division within common SerRS leading to Archaea is well supported under both maximum-likelihood and Bayesian models, and shows a broad and complex phylogenetic distribution implying HGT between many groups. Represented archaeal groups include Korarchaeota, Thaumarchaeota, and Crenarchaeota, which may contain a secondary HGT to Archaeoglobales. Other euryarchaeal groups represented include *Parvarcheum*, *Nanosalina*, Thermococcales, *Nanoarchaeum equitans*, Thermoplasmatales, Methanomicrobiales, and Methanocellales. Eukarya forms a well-supported clade grouping together with *Nanosalina*. Interestingly, the one major topological conflict between maximum-likelihood and Bayesian reconstructions is the placement of *N. equitans*, which, in the Bayesian reconstruction, groups outside of the Archaea, with the Thermus/Deinococcus group (see Additional file 2). Given the extent of HGT and orthologous displacement between lineages, while it seems clear cytosolic eukaryal SerRS was an acquisition from Archaea, the specific donor lineage cannot be identified. The deeper divisions within bacterial SerRS are poorly supported and show many short branches, making the relative placement of the archaeal branch and their common ancestor uncertain. As a further consequence, the position of the root within Actinobacteria in subtree A is almost certainly artifactual. However, there is still a major apparent division between bacterial and archaeal/eukaryal forms within the tree, near the placement of the root. For this reason, we consider it likely that the deep divisions within common SerRS phylogeny contain the node corresponding to LUCA.

In contrast, the rare SerRS group (B) is only represented by a subset of groups within the methanogens, including Methanosarcinales and at least one species of Methanomicrobiales, and another subgroup containing Methanococcales, Methanobacteriales, and Methanopyrales. The latter three orders represent the members of Group I methanogens, an ancient clade within Euryarchaeota. We propose that this distribution suggests an HGT from an ancient lineage into either (1) the methanogen ancestor, with subsequent back-transfers of the common type and orthologous displacements within some derived lineages, or (2) parallel or stepwise transfers into the ancestor of Group I methanogens and Methanosarcinales. In either case, additional secondary HGT events likely occurred into *Methanosphaerula* and at least one lineage related to Thermoplasmatales.

The placement of the node(s) corresponding to LUCA is less clear within the ThrRS family, although the deep placement of the rare type on a long branch is still suggestive of HGT from a pre-LUCA lineage. The taxonomic distribution of archaeal taxa within the rare form is broader than in SerRS; however, the well-supported phylogeny of the rare ThrRS group (D) suggests frequent HGT between these lineages. Therefore, the taxonomic distribution does not directly support the ancestor of the rare form being congruent with the archaeal ancestor node. The deepest group in the rare ThrRS tree groups most crenarchaeal lineages with Nanoarchaeota; however, the polarity of the hypothetical HGT resulting in this grouping cannot be determined, as other basal euryarchaeal lineages (Thermococcales) and crenarchaeal outgroups (Korarchaeota and Thaumarchaeota) appear to have been recipients of other HGT events and orthologous displacements for ThrRS. Therefore, it cannot be determined if this basal split within rare ThrRS reflects the Euryarchaeal/TACK division, the Thermococcales + Nanoarchaeota/methanogen division within Euryarchaeota, or independent transfers from an unknown lineage to either Nanoarchaeota or Crenarchaeota (followed by secondary HGT), and to methanogens. The methanogen group within rare ThrRS also lacks a clear signal of vertical inheritance, as the group I/group II methanogen division is not observed, with Methanosarcinales/Methanocellales/Thermococcales/Archaeoglobales constituting one group (together with at least one Thermoplasmatales representative, and *Cenarchaeum*), and group I methanogens together with Methanomicrobiales and some Halobacteriales constituting the other group. We conclude that the ancestor of this group is likely at least as old as the group I methanogens, but may not be as ancient as the methanogen ancestor lineage, and represent a more recent acquisition via HGT.

The common ThrRS group (C) consists of all bacterial and eukaryal ThrRS including some archaeal lineages via HGT. As is the case with SerRS, eukaryal ThrRS probably was acquired via HGT from within this group, although its *Parvarchaeum* sister within the tree obscures whether or not this was a primary HGT from bacteria, or a secondary event from an archaeal donor. Koarchaeota, *Nitrosopumilus*, *Parvarchaeum*, and some groups within Desulfurococcales, Methanomicrobiales, and Methanomassiliicoccales all apparently acquired common forms of HGT from within Bacteria. A poorly supported deep division within common ThrRS also contains a number of archaeal groups, including Halobacteriales, Sulfolobales, and Thermoplasmatales. Taken together, these archaeal common forms do not show the same broad distribution or cohesion as within SerRS, so that it is less clear that node (C) corresponds to LUCA. More generally, the Domain distributions within the rare archaeal (D) and common, mostly bacterial/eukaryal (C) forms of ThrRS are more similar to a post-LUCA division than those observed across SerRS forms. However, the stem branches separating these groups are long, and much more similar to the proposed pre-LUCA stem branches between (A) and (B) within SerRS, than between the bacterial and archaeal groups within the common SerRS form (A). This is even more apparent once poorly aligned sites are removed from the analysis, as described below. Therefore, the probability of a pre-LUCA divergence of rare ThrRS forms hinges to some extent on the evidence of a pre-LUCA divergence of rare SerRS forms. An ancestor of (D) and (C) congruent with LUCA is still a clear possibility, if evolution was much more rapid in both ThrRS Domain stem ancestors than in SerRS Domain ancestors within (A). A likely cause for such a change in evolutionary rates is not readily apparent.

### Functional divergence of rare aaRS forms

Deeply branching clades within a gene tree, such as that observed for the rare type SerRS, can also be evidence of an artifactual placement arising from rapid evolution of a lineage, resulting in long branch attraction (LBA). This is even more likely in cases where sequence divergence is accelerated via positive selection driven by the acquisition of novel function(s). Indeed, the N-terminal region of rare SerRS is entirely novel, as is its Zn-dependent mechanism for substrate binding, which is unique among aaRS [51]. However, we assert that these changes are far too fundamental to be explained by the hypothesis of gene duplication plus LBA alone, but require a much longer evolutionary history and a more distant shared origin, accessed via HGT. A radical within-lineage functional divergence would suggest the acquisition of a new function for the rare SerRS variant; however, this is unlikely to be the case, as after its introduction some lineages retained the ancestral form and lost the rare form, while other lineages kept the rare form and lost the ancestral form. This is even the case for closely related groups, such as the genus *Methanosarcina*: *M. acetivorans* and *M. mazei* both possess the common form of SerRS, while *M. barkeri* carries the rare form. This suggests functional equivalence, which is more consistent with an invasion followed by sorting. Even if the functional roles of the rare and common SerRS types were somehow different, they both still recognize the same cognate tRNA type, which is highly conserved. Therefore, the tRNA recognition domain should be under strong continuous purifying selection, and be least likely to change over such a short time, even if the rest of the protein is under positive selection to acquire novel function(s). In fact, tRNA recognition is under such strong purifying selection within aaRS proteins that HGT events between domains can even result in recombined displacements, with chimeric gene products retaining the vertically-inherited tRNA recognition domain [52]. Yet, in the case of SerRS the exact opposite scenario is observed, with the most radical difference between the rare and common form being the region involved in tRNA recognition. This suggests that the rare form coevolved in a distant lineage with a different tRNA, undergoing subsequent adaptations to accommodate the methanogen tRNA following transfer.

### Testing for LBA and artifactual placement of rare aaRS types

In order to test if the deep placement of rare SerRS forms is an LBA artifact arising from low sequence alignment quality or biases within phylogenetic reconstruction, phylogenetic analyses were performed using Bayesian reconstruction models more resistant to LBA effects, on sequence alignments maximized to include only well-aligned sites across clades informative for resolving the relationship between rare and common aaRS forms. This is especially important in the case of SerRS, which contains relatively few well-aligned sites between rare and common forms, and so may be especially sensitive to artifacts arising from alignment error.

### Alignment decomposition

Tree accuracy and resolution is generally improved by the masking of divergent sites within an alignment where homology across sequences is unlikely to be accurately inferred. This may contribute reconstruction errors, including LBA [53]. Several programs have been developed to detect and mask poorly aligned sites (e.g., [53-56]). Generally, these programs identify columns in an alignment that fail a statistical test, resulting in them being removed before phylogenetic analyses are performed. Some algorithms, such as AliGroove, also detect and mask poorly aligned sites at the individual taxon level [56]. However, as suggested by Wu et al. [55], these global column-specific approaches may mask well-aligned, phylogenetically informative sites within a particular clade, if other clades within the alignment have a poorer quality alignment for these sites.

While SerRS and ThrRS are homologous, they are phylogenetically distant, and so contain some regions that clearly align well between paralogs, and other regions that align more poorly, or may not even be homologous. Therefore, many sites that are well aligned *within* each paralog, and likely informative for determining the relationship between each rare and common form, may not reliabily align *between* paralogs. Determining the phylogenetic relationship between the rare and common forms of SerRS and ThrRS depends upon signal from both sets of sites. Therefore, to extend the observation of Wu et al. [55], clade-specific misalignment can also be a reciprocal problem which masking alone cannot solve. In order to surmount this, we employed a novel method of *alignment decomposition*. Using the masking of poorly aligned sites in GUIDANCE [54] we identified 107 sites as being well-aligned across SerRS and ThrRS paralogs. 129 additional sites were identified as well aligned specifically within the sub-alignment of SerRS, and 217 additional sites were identified as well aligned specifically within the sub-alignment ThrRS. Concatenating these mutually exclusive selections of sites produces a composite alignment that preserves a maximal amount of information for reconstructing the placement of the rare SerRS and ThrRS types (see Additional file 3).

### Bayesian phylogenetic reconstruction

Bayesian tree reconstruction methods taking into account site-specific equilibrium frequencies, such as PhyloBayes, are more robust against LBA artifacts [57]. A phylogenetic tree generated from the decomposed SerRS/ThrRS alignment using PhyloBayes returned a similar phylogeny to the maximum-likelihood tree, with the same deep placements for the rare SerRS and ThrRS forms, with high posterior probabilities (Figure 2, see Additional file 2). Mitigating these potential causes of LBA does not appear to perturb the deep placement of the rare forms within the phylogeny, supporting that this placement is not artifactual. The improved overall signal of the GUIDANCE-decomposed, PhyloBayes tree is evident in the recovery of many of the same deep splits within each aaRS form as the maximum-likelihood tree, but with much higher support, even on very short internal branches. For example, in the common type SerRS, a major bacterial grouping including members of Thermotogales, Aquificales, Cyanobacteria, Spirochaetes, Proteobacteria, Clostridia, Bacilliales, and some *Methanosarcina* species is on a very short branch with a low maximum-likelihood bootstrap support of 20; this same clade is recovered in the PhyloBayes reconstruction, with a posterior probability of 0.89. Similar increased supports are observed within ThrRS, with a bacterial grouping of Aquificales, Alphaproteobacteria, Chloroflexi, and Actinobacteria having a low bootstrap support of 33, and a posterior probability of 0.98 in the PhyloBayes reconstruction.

### Character site analysis

Another means of testing the LBA hypothesis within the rare types of SerRS and ThrRS is by observing the frequency of character sites within amino acid sequence alignments. At slowly evolving positions, these sites support a particular bipartition within a tree by recording a single change that occurred along that bipartition. Within a rooted tree, character sites can reflect either shared ancestral characters (symplesiomorphies) or derived characters (synapomorphies). If the deep placements of rare forms of SerRS and ThrRS are artifactual, symplesiomorphies for these groups with the opposing aaRS sister family should not be observed, as the true placement within the common type of each family would preclude the existence of the required bipartition. In the case of an LBA artifact, any apparent symplesiomorphies would need to be explained by convergent evolution along the long branch leading to the rare form emerging from within the common forms. As these convergent sites should be very rare, the LBA hypothesis can be evaluated, in each case, by comparing the number of observed symplesiomorphy character sites between the rare and common forms of each aaRS. Additionally, it is expected that the counts of character sites within rare and common types should be proportional to stem branch lengths to each group; consequently, symplesiomorphies should depend on the length of the sister clade stem branch. No such relationship is expected for the frequency of observed convergent sites, in the case of an LBA artifact. We find that the number of symplesiomorphic character sites found in the rare SerRS type (3) is comparable to that observed for the common SerRS type (4), supporting true deep ancestry rather than LBA as an explanation for the placement of these rare forms (Table 1). All of these symplesiomorphic sites show radical AA differences between rare and common SerRS forms, further arguing against homoplasy. In the case of sites within ThrRS, no strong symplesiomorphies were observed for rare ThrRS. Only a single weak site was observed (580D). This site shows low sequence conservation within common ThrRS, and does not contain D. While nearly all of the common SerRS also contain a D at this site, only a few rare SerRS taxa contain a similar amino acid (E), with others showing a diverse set of generally hydrophobic residues. Nevertheless, this distribution suggests D is the ancestral form with SerRS/ThrRS, retained within the rare ThrRS lineage. The low count of rare ThrRS synapomorphies is likely due to the much shorter branch leading to the ThrRS common forms, making substitutions at slow-evolving sites less likely along this branch (and, consequently, making rare ThrRS symplesiomorphies unlikely).

**Table 1:** **Similar counts of shared ancestral sites for rare and common forms of SerRS support a non-artifactual deep placement of rare SerRS**

**Symplesiomorphies**	**Conserved residues (%)**				
	**Site**	**Common SerRS**	**Rare SerRS**	**Common ThrRS**	**Rare ThrRS**
Rare SerRS	598	G(65)	P(89)	P(86)	P(100)
	758	R/K(80)	G(100)	G(98)	G(100)
	995	L/M(64)	F(100)	F(80)	F(64)
Common SerRS	568	H(98)	E(56)	H(98)	H(83)
	994	R(88)	G(94)	R(89)	R(97)
	1082	V(71)	not conserved*	V(86)	E(83)
	1101	E(99)	A(83)	E(100)	E(83)

Physiochemically similar amino acids were grouped together if both were distinct from the alternative AA residue, and the subsequent shared count exceeded 50%. See methods for detailed character site definitions. *sites include E(44%) and K(22%), distinct from alternative residue (V), but not physiochemically conserved.

### Ancient origin of the two divergent forms of glycyl-tRNA synthetases (GlyRS)

**Figure 3:**
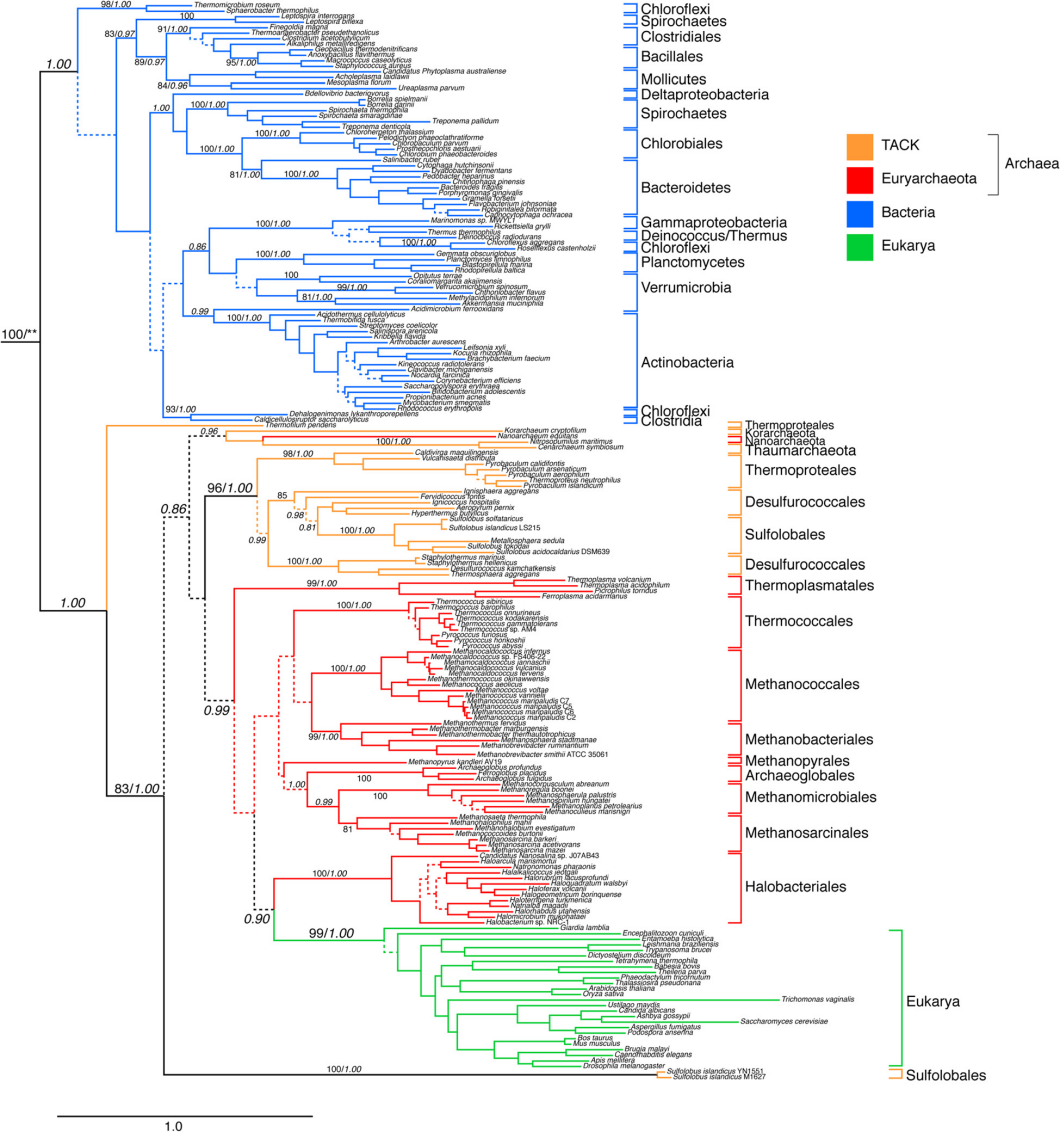
ML tree of the GlyRS homodimer subunit. Branch colors depict Bacteria (blue), *Euryarchaeota* (red), *Crenarchaeota* (green) and Eukarya (magenta). More narrow taxonomic designations are bracketed and labeled. Dotted lines represent branches with <50% BS support; major clades with >80% BS support are labeled with respective BS support values. Italic numbers represent posterior probability (PP) support values from tree reconstruction in PB. The GlyRS homodimer alignment contains 199 taxa, an alignment length of 1344 amino acids and rooted using sequences from ProRS and SerRS of *Escherichia coli* and *Methanosarcina acetivorans* (not shown). For the PB reconstruction, a subset of the alignment was used, consisting of 400 well-aligned sites as identified by GUIDANCE. Branch lengths depict substitutions/site. (**)PP support for the placement of the root was not calculated under PB, which failed to converge when outgroups were included. Non-converged PB runs including outgroups placed the outgroup along the same branch as the ML tree.

**Figure 4:**
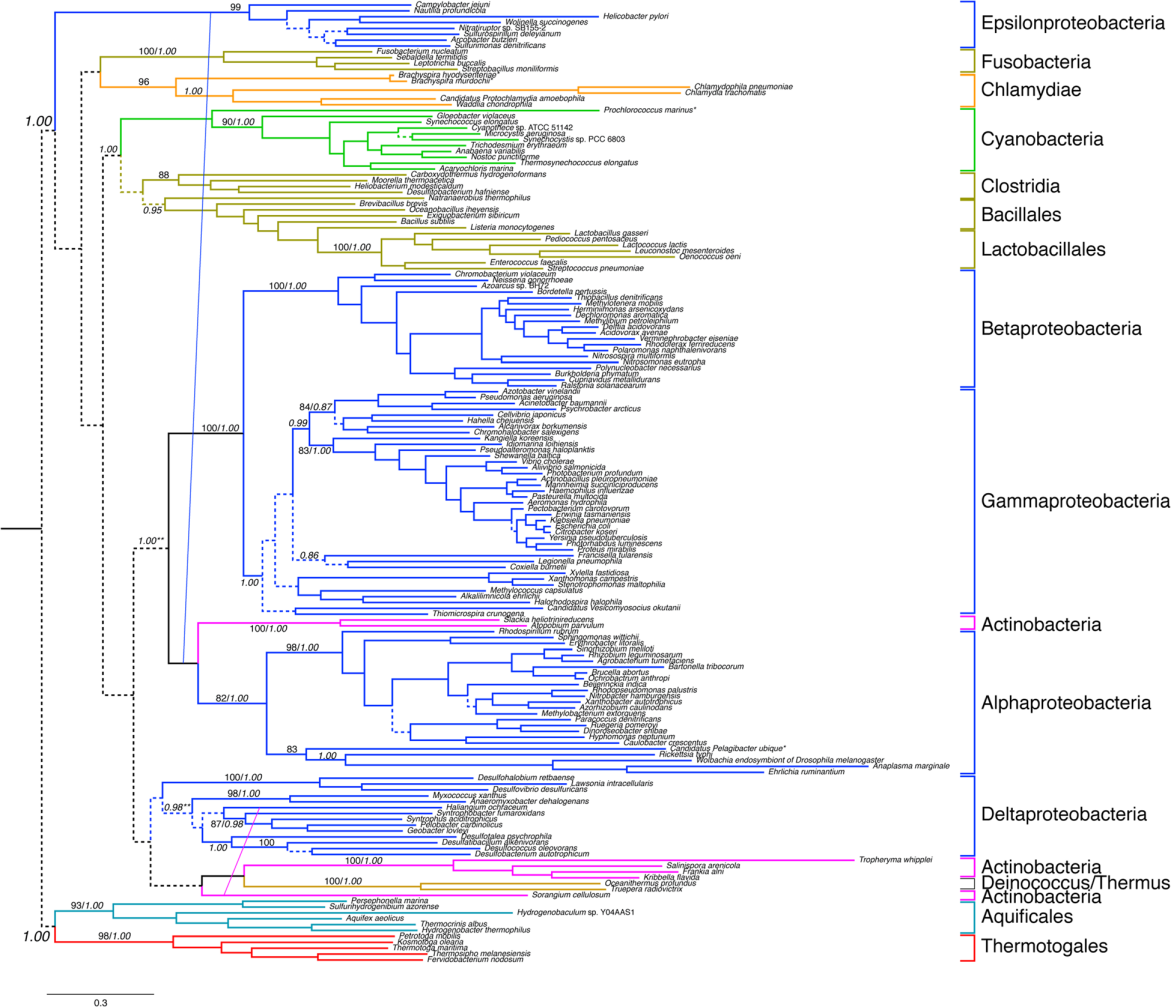
Phylogenetic analyses of the GlyRS heterodimer subunits. Tree represents history of concatenated sequences of the α_2_ and β_2_ subunits of the heterotetramer form. This form is found only in the bacterial domain. Colors specify major bacterial clades, including Proteobacteria (blue), Actinobacteria (magenta) and Firmicutes (brown). More narrow taxonomic designations are bracketed and labeled. Dotted lines represent branches with <50% BS support; major clades with >80% BS support are labeled with respective BS support values. Italic numbers represent posterior probability (PP) support values from tree reconstruction in PB. For the PB reconstruction, a subset of the alignment was used, consisting of 743 well-aligned sites identified by GUIDANCE. Significant conflict between the ML and PB trees is shown by an alternative diagonal branching. The alignment contains 168 taxa with alignment length of 2028 amino acids. The tree was rooted placing Thermotogales/Aquifex as the outgroup (see methods for rooting details)*.* Branch lengths depict substitutions/site. (*) long-branched taxa removed in the PB analysis to facilitate convergence. (**)PP support value in the alternative PB topology.

GlyRS catalyzes the addition of the amino acid glycine to its cognate tRNA. Two distinct forms of GlyRS exist - an α_2_ homodimer found in all Archaea, Eukarya and in some bacterial phyla (Figure 3) and an α_2_β_2_ heterotetramer distributed in the majority of the Bacteria (Figure 4). The homodimeric type shows a clear phylogenetic signal of a major bifurcation between bacterial and archaeal/eukaryal forms, weakly supported in the maximum-likelihood reconstruction, and strongly supported in the GUIDANCE-PhyloBayes reconstruction (see Additional file 4). We identify this primary bifurcation as corresponding to LUCA. While many internal branches within each domain are poorly supported under both reconstructions, many strongly supported groups reflect well-established clades, such as Chlorobiales/Bacteroidetes, Crenarchaeota, and Euryarchaeota. There are also several clear instances of HGT events, such as from within Desulfurococcales to the ancestor of Sulfolobales. Eukarya and Halobacteriales are sister clades, strongly supported under the GUIDANCE-PhyloBayes reconstruction, and indicating an HGT event. However, as this group is placed deeply within Euryarchaeota and does not have a well-supported outgroup, the direction of HGT cannot be clearly established. It seems more likely that this reflects an HGT from the halobacterial lineage to the eukaryal ancestor; however, the evolutionary history could also include multiple, possibly secondary HGTs from more deeply branching donor(s).

The concatenated heterodimer subunit sequences of the GlyRS heterotetramer form also produce phylogenies indicative of an ancient origin within Bacteria. While alignment to outgroup sequences is unreliable, both maximum-likelihood and GUIDANCE-PhyloBayes reconstructions support Thermotogales and Aquificales as sister clades (see Additional file 5). In the case of the GUIDANCE-Phylobayes reconstruction, this relationship is strongly supported with a posterior probability of 1.00. Generally, these groups are only adjacent when in basal positions within phylogenetic reconstructions, as the alternative placement of Thermotogales is typically among Firmicutes, and the alternative placement of Aquificales among Epsilonproteobacteria [58]. The distribution of the heterotetramer form includes most Proteobacteria, as well as representatives of many other bacterial phyla including Cyanobacteria, Clostridia, and Bacillales/Lactobacillales. While the maximum-likelihood topology places Epsilonproteobacteria deeply within the tree and apart from other Proteobacteria, the GUIDANCE-PhyloBayes reconstruction groups them together with Beta-, Gamma, and Alpha-proteobacteria, with a high posterior probability of 1.00. Deltaproteobacteria (along with representitives of some other bacterial phyla) are a sister to this group, although there is low support for proteobacterial monophyly (with subsequent HGT to within Actinobacteria and Deinococcus/Thermus). Taken together, this suggests that the heterotetrameric form is ancestral to the bacterial domain, has been vertically inherited and retained in many lineages, and has been lost and/or transferred to many others.

The two forms of GlyRS differ significantly in their sequence and structure. In some cases, a particular phylum or class may consist of members carrying one or the other form of GlyRS, as in the case of Actinobacteria, Spirochaetes, Firmicutes, Gammaproteobacteria and the Thermus-Deinococcus group. Independent evolution of two GlyRS systems would have precedent, given the emergence of two LysRS from different aaRS classes [34,59-61]. However, in contrast to LysRS, the two GlyRS have shared ancestry, as both are members of the Class II family. Furthermore, tRNA^Gly^ recognition has been conserved for both enzymes [62,63]. Homology between the two forms is masked by large structural differences between them. The α_2_ form is in the same subclass as ProRS, SerRS and ThrRS (subclass IIa), while the GlyRS heterotetramer groups with PheRS (subclass IIc), another α_2_β_2_ heterotetramer enzyme [36]. Therefore, although these forms share a common ancestor, GlyRS protein families are likely polyphyletic. It has been reported that the β subunit of the GlyRS heterotetramer showed significant similarity to the HD superfamily of hydrolases [61], and plays a significant role in tRNA recognition [64]. Also, some additional genes [65,66] and other isofunctional enzymes [67] may be true homologs of GlyRS, but the extremely long period of time and fast rate of evolution since their divergence may have concealed their common ancestry, which is not clearly apparent in sequence-based analysis.

The duplications that gave rise to members of each aaRS class already had occurred by the time of LUCA, as evident from the clustering of the enzymes based on substrate specificity and not on species identity [35]. Hence, the origin of the two GlyRS forms, despite significant differences in structure, can be traced back to a pre-LUCA ancient ancestor within the Class II aaRS family. Because the two GlyRS belong to different subclasses, their common ancestor is even more ancient than that of the two different forms of SerRS and ThrRS, which are both members of the subclass IIa. Therefore, the specificity of the two GlyRS families may be either an instance of convergent evolution, or possibly even reflect the ancestral specificity of all Class II aaRS. The sporadic distribution of the archaeal GlyRS in Bacteria and the presence of one or the other form in a number of bacterial phyla suggests that HGT played an important role in its evolution, specifically after the extant bacterial phyla came into existence. One scenario for the evolution of this enzyme is that an ancestral GlyRS α subunit gave rise to the two extant GlyRS α subunit types in each GlyRS variant. In this scenario, one variant became the constituent of the homodimer, and the other became a component of the heterotetramer. Because the homodimer is distributed in all three domains, it has been suggested that this is the more ancient form [60,61]. However, given the deep relationship of the heterodimeric subunits to other class II aaRS, and their likely ancestral presence in the Bacterial Domain, distribution alone is not a sufficient criterion to determine the temporal ordering of these types. An HGT of the heterodimer subunits from an ancient, extinct lineage to the bacterial Domain ancestor would result in the same distribution, as well as explain the deep rooting of this group within the class II phylogeny.

The question remains as to why different taxonomic groups appear cohesive and monophyletic within both GlyRS trees, which would initially suggest that the evolution of GlyRS is dominated by vertical inheritance, in contrast with other aaRS where HGT is commonplace. However, both vertical inheritance and high frequency of within-group HGT can result to the same evolutionary pattern [68]. In both Gly trees, there are several very basal bacterial relationships that appear to be maintained, likely indicating deep vertical inheritance. For example, Bacteroidetes/Chlorobi, most Firmicutes/Mollicutes and most Proteobacteria group together within these phylogenies. However, HGT remains an important process in GlyRS evolution, as different groups appear to have inherited both forms of the enzyme, with a resulting distribution that does not seem to be the result of lineage-specific duplication and sorting events. We propose that the heterotetramer form of GlyRS in Bacteria was likely ancestrally horizontally acquired from outside the group. Subsequent HGTs between the archaeal ancestor and Bacteria, and between bacterial clades then resulted in the differential inheritance of each GlyRS form. These same processes of ancient HGT from extinct ancestors may also have provided a divergent, deeply branching homodimer form to *Thermofilum pendens*. Even more striking is the even more deeply branching form of the GlyRS homodimer found within some strains of *Sulfolobus islandicus*; other strains of *S. islandicus* contain a homodimeric form that groups with other members of Sulfolobales within Crenarchaeota. This suggests that a reservoir of divergent aaRS forms must still exist, facilitating these transfers to even very recently diverged groups; future environmental sequencing efforts will likely discover similar cases, and possible donor groups for these cryptic transfers.

The ancient, pre-LUCA origins of these enzymes imply that at the time of the last organismal ancestor, the translation machinery was already in place, albeit likely somewhat different than what exists today. Rare and unusual aaRS and other relic genes with similar histories may be some of the only evidence that remains of extinct sister lineages of LUCA, and the earliest cellular ecosystems.

## Conclusions

### A plurality of ancestors

The origin of life did not coincide with the organismal LUCA; rather, a profound gap in time, biological evolution, geochemical change, and surviving evidence separates the two. After life emerged from prebiotic processes, diversification ensued and the initial self-replicating and evolving living systems occupied a wide range of available ecological niches. From this time until the existence of the organismal LUCA, living systems, lineages and communities would have come and gone, evolving via the same processes that are at work today, including speciation, extinction, and gene transfer. Analogous to the rapid innovation of animal body plans during the Cambrian explosion, perhaps many of the early, possibly exotic forms of life did not give rise to extant lineages [69,70].

Several studies have reported a definable phenotype of the organismal ancestor of all known life. It is estimated that LUCA possessed 500–600 genes, and that cellular physiology was already very complex by this time [71]. This ancestor was already metabolically and genetically similar to modern cells. It already possessed functional capabilities for transcription, replication, translation, ATP synthesis, chemiosmotic coupling, signal recognition, and assimilation of amino acids and nucleotides [72,73]. Another study that supports this concept of a modern-like ancestral cell proposed that the organismal LUCA possessed a lipid membrane, based on the phylogenetic analyses of two dehydrogenases, an archaeal G1PDH and a bacterial/eukaryal G3PDH [74]. Given the genetic and functional complexity of this ancestral LUCA organism, it seems necessary that a long period of time separated the first life on earth and the emergence of the cell from which extant life diverged. During this interval, the consequences of Darwinian evolution observed in organisms today would also exist, leading to speciation and divergence of descendant lineages containing biological diversity. When the cell ancestral to all extant life appeared, it was not the only existing cell at that time; a wide variety of cellular entities existed prior to and during the organismal LUCA era. LUCA itself may not even have been “special” in any way, but an arbitrary point of coalescence [49].

### Hypnologs: genes from lost lineages

During the time of LUCA, microbes probably interacted with each other and inhabited different niches, the same way organisms do today. It is reasonable to assume that these organisms also transferred genetic material with each other, allowing metabolic inventions to spread rapidly in the biosphere. Through HGT organisms are tied together into larger groups of exchange, greatly accelerating the speed of evolution [75]. Without genetic exchange, innovations have to occur successively in the same lineage; with genetic exchange, innovation made in different lineages can be brought together in a singe cell. Although phylogenetic reconstruction of genes involved in such ancient transfers would show that each gene emerged from a singular common origin, (the last universal gene ancestor, or using Walter Fitch’s terminology, the gene-cenancestor [76]), there is no reason to assume that the cenancestors of all genes were at one time present in the same lineage representing the cellular ancestor of all extant life. While an early mass extinction with a lone survivor lineage representing LUCA would collapse each gene ancestor onto the organismal ancestor lineage, we infer lineages co-existing with LUCA, arguing against such a narrow bottleneck. It is more reasonable to assume that many ancient genes have been acquired from lineages branching more deeply than the organismal ancestor lineage of the three domains of life. Subsequent vertical inheritance of these transferred genes would propagate them to extant lineages, even if the donor lineages have gone extinct.

**Figure 5:**
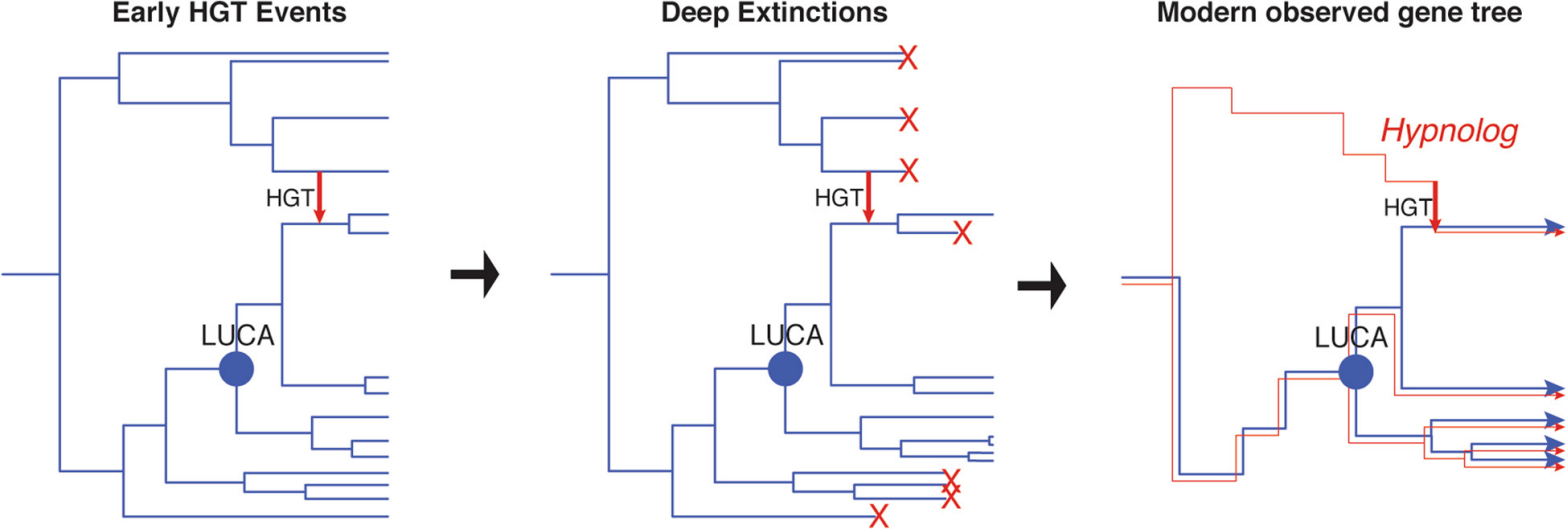
Hypnologs. HGT from ancient lineages that diverge before LUCA into the ancestors of extant lineages produce a unique phylogenetic signal within gene families. Species trees (blue) become pruned over time via extinction, while gene ancestry (red) from extinct lineages can be preserved by the HGT event, resulting in a gene ancestor predating the LUCA species ancestor.

As may be the case for the aaRS genes discussed here, these ancient transfer events from lineages diverging pre-LUCA could have continued occurring as long as these lineages persisted, even after divergences of extant groups after the time of LUCA. This novel kind of HGT can be generalized to other more recently extinct lineages within any given phylogeny, where the extinct lineage is a member of an unclassified and unsampled outgroup. As this evolutionary scenario produces unique consequences for phylogenetic inference, we propose a new term to describe these transferred genes, “hypnologs” (Figure 5). This term is derived from the name of the mythological Greek god Hypnos, from whose cave in the underworld flowed Lethe, the river of forgetfulness and oblivion. Therefore, the term succinctly captures the concept of the flow of genetic information into and along lineages, death/extinction, and the resulting loss of evolutionary history and phylogenetic information that generates the peculiar sparse, deep phylogenetic distributions of affected genes.

HGT events provide important clues about the early evolution of life on Earth, even before LUCA. Reticulations in the Net of Life due to HGT are not restricted to recent transfers but have occurred throughout life’s history. For ancient transfers, especially those that took place before the divergence of the two prokaryotic domains during the time of LUCA, it becomes more challenging to trace the history of transferred genes in part because the donor organism’s lineage might have gone extinct since the transfer took place. In fact, major catastrophic events early in Earth’s history argue for such massive microbial extinctions, including impacts (even possibly during the Late Heavy Bombardment, or similar Hadean/Archaean major impact events) or the Great Oxygenation Event. However, through HGT, some of their genetic material may still persist as an evolutionary relic of ancient extinct lineages. Patterns in the phylogeny and distribution of atypical aaRS proteins provide insight into these processes, revealing their importance in explaining the observed patterns of gene inheritance within extant lineages.

## Methods

### Sequence collection, alignments and BLAST

Amino acid sequences of SerRS, ThrRS, and GlyRS were collected from GenBank [77]. Sequences were selected from members of major taxonomic groups at the level of kingdom (Eukarya) phylum (Bacteria) and order (Archaea) within the three domains of life, in order to provide a representative sampling to a depth informative for this study. For the PylS analysis, all available PylS sequences within GenBank were used. This was confirmed with BLAST searches showing that all hits to additional aaRS sequences were all more closely related to other annotated aaRS families. For SerRS, ThrRS and GlyRS analyses, a profile alignment was performed by initially aligning the rare and common forms of each enzyme separately. The alignments of the rare and common forms were then aligned to one another as profiles. In the case of SerRS and ThrRS, the respective protein family alignments were subsequently aligned in an additional profile alignment step. The sequences of the α_2_ and β_2_ subunits of the heterotetramer GlyRS were initially aligned separately. Trees reconstructed from the individual α_2_ and β_2_ subunit alignments are similar in terms of the clustering of the taxa at the level of phylum and order, suggesting these are co-inherited. Therefore, in order to maximize phylogenetic resolution, subsequent phylogenetic analyses were performed on a concatenated alignment of the two subunits. For PylRS, the N-terminal region of the protein sequence corresponding to the N-terminal split gene product in Bacteria was not used in the phylogenetic reconstruction, as this region is absent within Methanomassiliicoccales. All sequences were aligned using MUSCLE [78] with default parameters. Alignment sequences are available as supporting information (see Additional files 6, 7, 8 and 9).

### Selection of well-aligned alignment sites

For GlyRS and SerRS multiple sequence alignments, the GUIDANCE program was used to identify and remove blocks of poorly aligned sites (default parameters) (see Additional files 10 and 11) [54]. GUIDANCE analysis of PylRS revealed a high-quality alignment without need of column removals. Poorly aligned regions of outgroup sequences for PylRS and homodimeric GlyRS alignments were removed. The additional step of alignment decomposition was implemented for SerRS/ThrRS GUIDANCE results.

### Alignment decomposition

For the SerRS/ThrRS alignment, GUIDANCE identified 107 sites as being well-aligned across SerRS and ThrRS paralogs. 129 additional sites were identified as well aligned specifically within the sub-alignment of SerRS, and 217 additional sites were identified as well aligned specifically within the sub-alignment ThrRS. These exclusive sets of sites were concatenated to produce a decomposed alignment with a total of 453 sites, with 236 sites informative for the phylogenetic relationship between rare and common SerRS forms, and 324 sites informative for the phylogenetic relationship between rare and common ThrRS forms.

### Tree reconstructions

We selected the best-fitting model of amino acid replacement for each aligned dataset based on the Akaike Information Criterion (AIC) using ProtTest [79]. Phylogenies and 100 bootstrap supports for all datasets were calculated with PhyML v3.0 [80] with the LG [81] amino acid substitution model, estimated portions of invariable sites, estimated Γ distribution parameter, four rate categories, estimated amino acid frequencies, and an NJ starting tree. We used the subtree pruning and regrafting (SPR) tree search method for PylRS because of the smaller number of sequences included (n = 37) and the faster nearest neighbor interchange (NNI) method for the much larger GlyRS homodimer, GlyRS heterotetramer and SerRS/ThrRS datasets. Support for nodes was assessed using 100 bootstrap replicates.

Bayesian phylogenetic inference was performed on all GUIDANCE alignment datasets and the PylRS alignment dataset using PhyloBayes3.3 [57] with the LG amino acid substitution model. For each analysis the CAT model was specified with fixed C60 site profiles. Chains in PhyloBayes3.3 were run until convergence (maxdiff ≤ 0.3, all effective parameter sizes ≥ 50, all parameter maximum discrepancies ≤ 0.3). Outgroups were excluded for the GlyRS homodimer alignment to facilitate run convergence, and from the GlyRS heterodimer alignment due to the poor quality of alignment of outgroups to ingroup sequences. Selected long-branched sequences were also excluded from the GlyRS heterodimer alignment to faciliate run convergence, as described in Figure 4. Tree topologies and posterior probability node support values were generated from a sample size of 100 trees following a 20% burn-in.

### Character site detection

A relaxed character site definition was used for identifying symplesiomorphies (shared ancestral character sites) for rare and common aaRS groups. A relaxed definition permits inclusion of residues that may not be completely conserved within a group, as derived lineages may experience additional substitutions at a site. Symplesiomorphic sites were identified as alignments sites for which >50% of taxa within an aaRS type ingroup have the same residue as >50% of the aaRS type outgroup, with this same residue being absent within all members of the sister group. For example, site 598 was identified as a symplesiomorphy within rare type SerRS, as nearly all of the rare SerRS contained the same amino acid as nearly all ThrRS sequences (P), while P was absent at this site within all common SerRS sequences, the majority of which contain G. All identified symplesiomorphy sites showed amino acid conservation levels much higher than the 50% threshold (the average SerRS-type conservation level was 92%), suggesting that this result is not impacted by stringency of the selected cutoff.

### Availability of supporting data

The data sets supporting the results of this study are available in the Dryad repository, doi:10.5061/dryad.hq4gc [81].

## Additional files

Additional file 1:
**Phylogenetic tree of PylRS reconstructed using PhyloBayes3.3.** Phylogenetic reconstruction of PylRS sequences from the full FASTA alignment, using PhyloBayes3.3 with C60 fixed CAT site profiles. Additional file 2:
**Phylogenetic tree of SerRS/ThrRS using GUIDANCE decomposed alignment, reconstructed using PhyloBayes3.3.** Phylogenetic reconstruction of SerRS/ThrRS sequences from the GUIDANCE decomposed FASTA alignment (Additional file 3) using PhyloBayes3.3 with C60 fixed CAT site profiles. Additional file 3:
**GUIDANCE decomposed alignment of SerRS/ThrRS.** Decomposed alignment of sites identified by GUIDANCE as being well-aligned between and within SerRS and ThrRS paralogs (see methods). Additional file 4:
**Phylogenetic tree of GlyRS homodimer using GUIDANCE alignment, reconstructed using PhyloBayes3.3.** Phylogenetic reconstruction of GlyRS homodimer sequences from the GUIDANCE FASTA alignment (Additional file 10) using PhyloBayes3.3 with C60 fixed CAT site profiles. Additional file 5:
**Phylogenetic tree of GlyRS heterodimer using GUIDANCE alignment, reconstructed using PhyloBayes3.3.** Phylogenetic reconstruction of GlyRS heterodimer sequences from the GUIDANCE FASTA alignment (Additional file 11) using PhyloBayes3.3 with C60 fixed CAT site profiles. Additional file 6:
**Aligned SerRS and ThrRS sequences.** MUSCLE-aligned rare and common forms of SerRS and ThrRS protein sequences sampled across 3 Domains of life. Additional file 7:
**Aligned GlyRS homodimer sequences.** MUSCLE-aligned of GlyRS homodimer subunit protein sequences sampled across 3 Domains of life, including other aaRS outgroups. Additional file 8:
**GlyRS heterodimer subunit sequences.** Concatenation of MUSCLE-aligned GlyRS heterodimer subunit protein sequences sampled across bacterial taxa, including other aaRS outgroups. Additional file 9:
**Aligned PylRS sequences.** MUSCLE-aligned C-terminal region homologous to C-terminal subunit in bacteria, including other aaRS outgroups. Additional file 10:
**GUIDANCE selected sites of GlyRS homodimer alignment.** Sites from Additional file 7 identified by GUIDANCE as being well-aligned, with single-taxon insertions removed. Additional file 11:
**GUIDANCE selected sites of GlyRS heterodimer alignment.** Sites from Additional file 8 identified by GUIDANCE as being well-aligned, with single-taxon insertions removed.

## Abbreviations

HGT: Horizontal gene transfer
LUCA: Last universal cellular/common ancestor
aaRS: Aminoacyl-tRNA synthetase
SerRS: Seryl-tRNA synthetase
ThrRS: Threonyl-tRNA synthetase
PylRS: Pyrrolysyl-tRNA synthetase
SepRS: O-phosphoseryl-tRNA synthetase
PheRS: Phenylalanyl-tRNA synthetase
GlyRS: Glycyl-tRNA synthetase
