# Supplementary figures and images for: Ancient horizontal gene transfer and the last common ancestors
Source: BMC Evol Biol. 2015 Apr 22;15:70. doi: 10.1186/s12862-015-0350-0 (PMC4427996; doi:10.1186/s12862-015-0350-0)

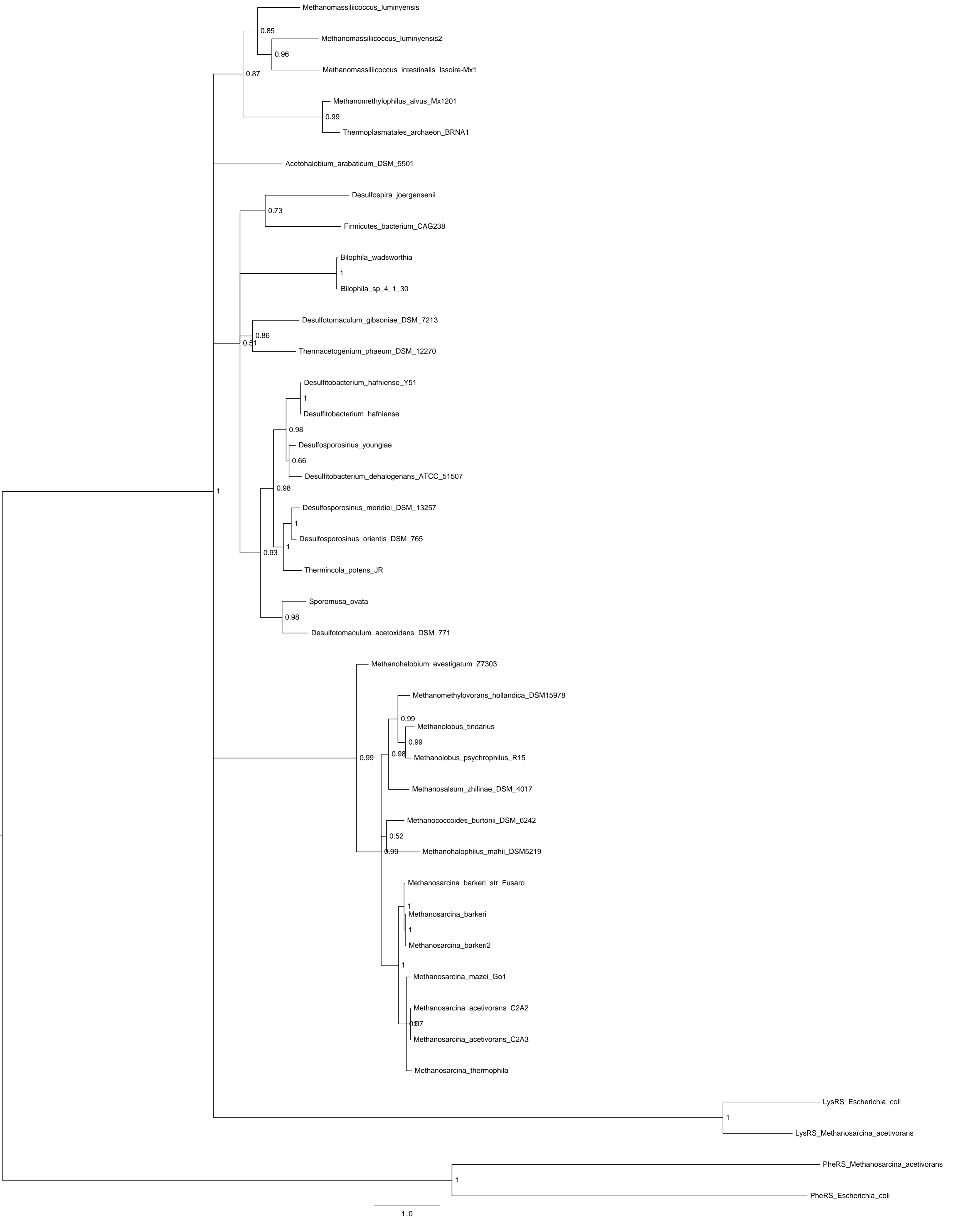

Supplement: Additional file 1: — Phylogenetic tree of PylRS reconstructed using PhyloBayes3.3. Phylogenetic reconstruction of PylRS sequences from the full FASTA alignment, using PhyloBayes3.3 with C60 fixed CAT site profiles. [file 12862_2015_350_MOESM1_ESM.pdf]

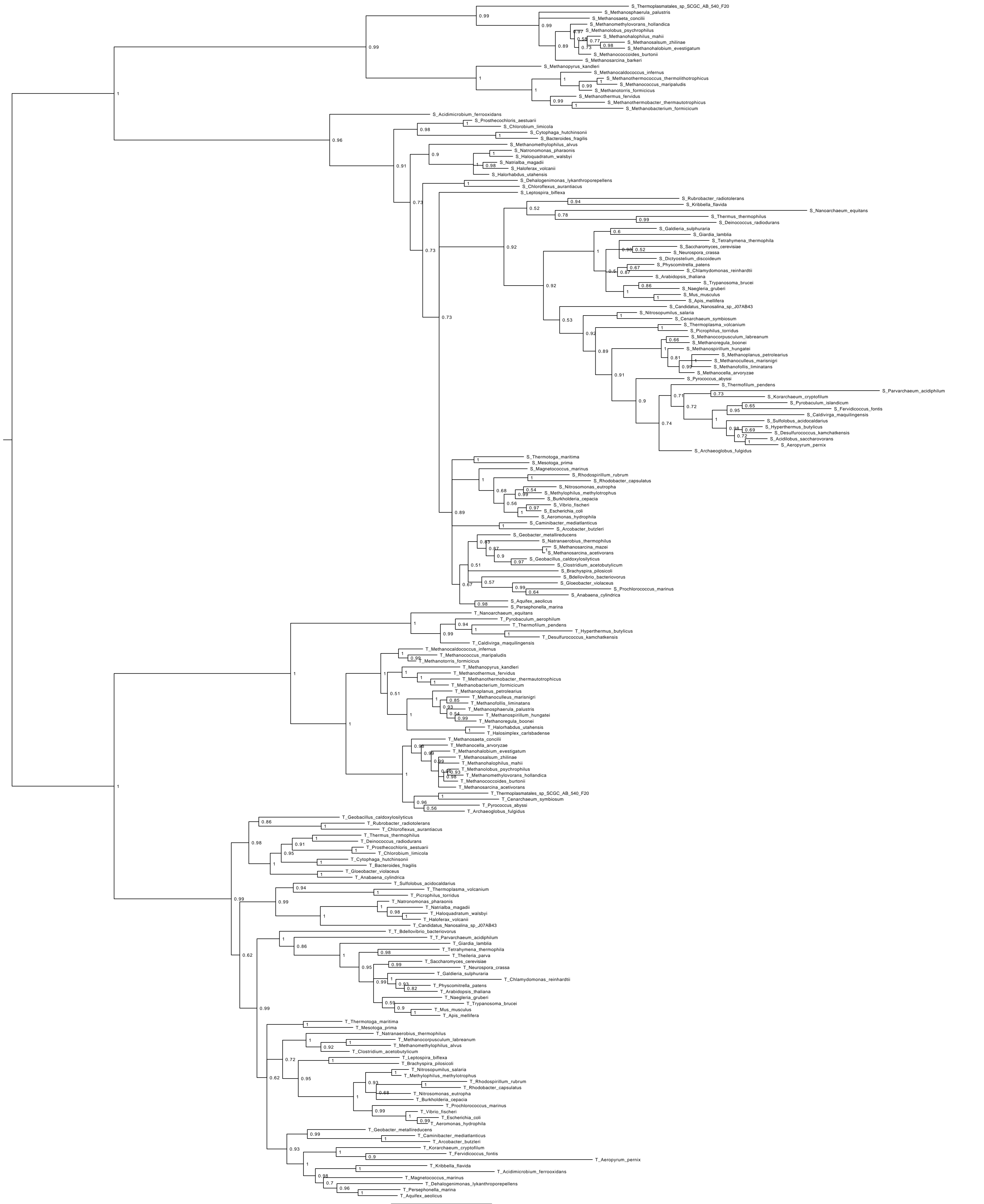

Supplement: Additional file 2: — Phylogenetic tree of SerRS/ThrRS using GUIDANCE decomposed alignment, reconstructed using PhyloBayes3.3. Phylogenetic reconstruction of SerRS/ThrRS sequences from the GUIDANCE decomposed FASTA alignment (Additional file 3) using PhyloBayes3.3 with C60 fixed CAT site profiles. [file 12862_2015_350_MOESM2_ESM.pdf]

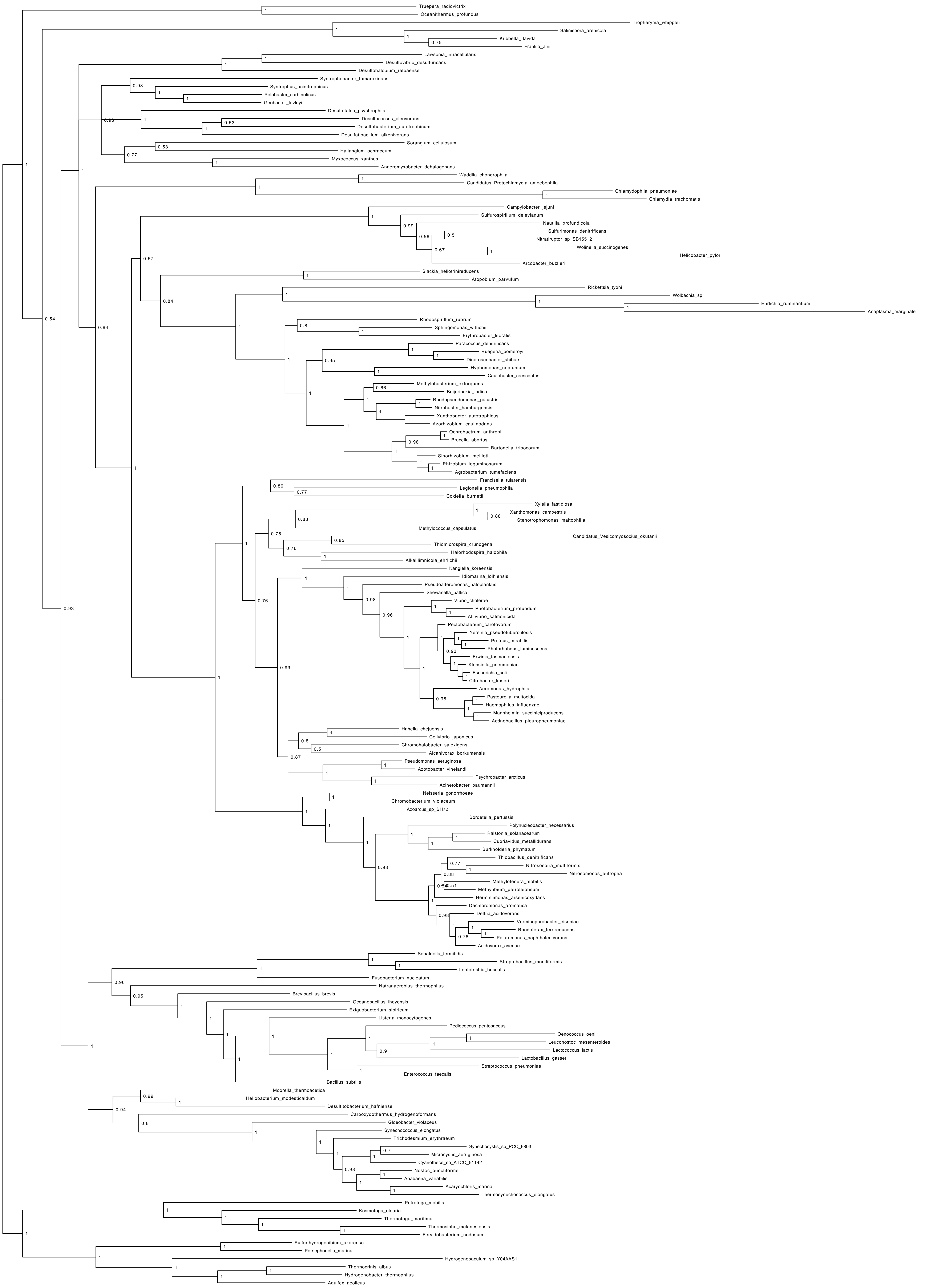

Supplement: Additional file 5: — Phylogenetic tree of GlyRS heterodimer using GUIDANCE alignment, reconstructed using PhyloBayes3.3. Phylogenetic reconstruction of GlyRS heterodimer sequences from the GUIDANCE FASTA alignment (Additional file 11) using PhyloBayes3.3 with C60 fixed CAT site profiles. [file 12862_2015_350_MOESM5_ESM.pdf]
