# Supplementary material for: Ancient horizontal gene transfer and the last common ancestors
Source: BMC Evol Biol. 2015 Apr 22;15:70. doi: 10.1186/s12862-015-0350-0 (PMC4427996; doi:10.1186/s12862-015-0350-0)
Supplement: Additional file 4: — Phylogenetic tree of GlyRS homodimer using GUIDANCE alignment, reconstructed using PhyloBayes3.3. Phylogenetic reconstruction of GlyRS homodimer sequences from the GUIDANCE FASTA alignment (Additional file 10) using PhyloBayes3.3 with C60 fixed CAT site profiles. [file 12862_2015_350_MOESM4_ESM.pdf]

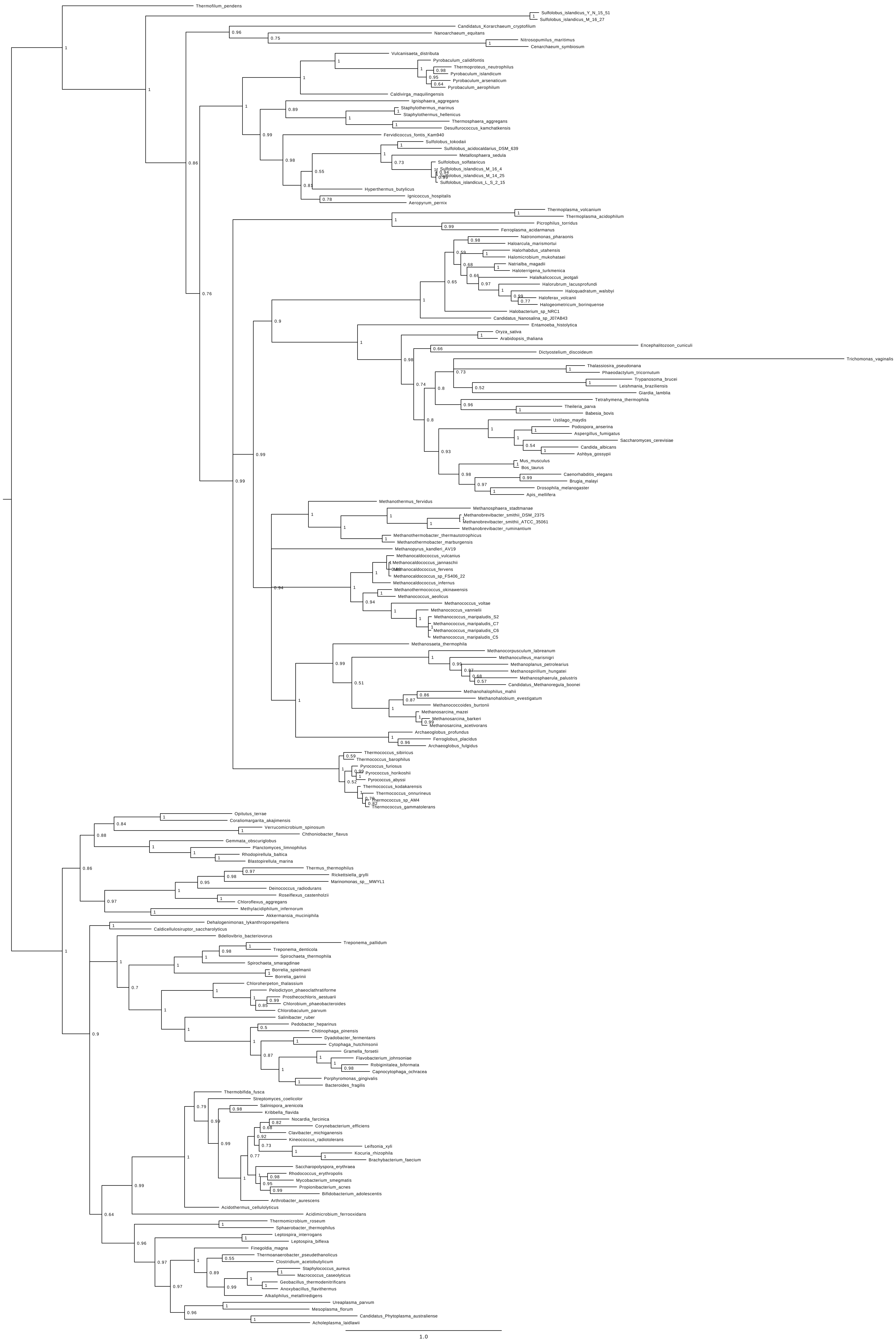

Thermofilum_pendens
Sulfolobus_islandicus_Y_N_15_51
Sulfolobus_islandicus_M_16_27
Candidatus_Korarchaeum_cryptofilum
Nanoarchaeum_equitans
Nitrosopumilus_maritimus
Cenarchaeum_symbiosum
Vulcanisaeta_distributa
Pyrobaculum_calidifontis
Thermoproteus_neutrophilus
Pyrobaculum_islandicum
Pyrobaculum_arsenaticum
Pyrobaculum_aerophilum
Caldivirga_maquilingensis
Ignisphaera_aggregans
Staphylothermus_marinus
Staphylothermus_hellenicus
Thermosphaera_aggregans
Desulfurococcus_kamchatkensis
Fervidicoccus_fontis_Kam940
Sulfolobus_tokodaii
Sulfolobus_acidocaldarius_DSM_639
Metallosphaera_sedula
Sulfolobus_solfataricus
Sulfolobus_islandicus_M_16_4
Sulfolobus_islandicus_M_14_25
Sulfolobus_islandicus_L_S_2_15
Hyperthermus_butylicus
Ignicoccus_hospitalis
Aeropyrum_pernix
Thermoplasma_volcanium
Thermoplasma_acidophilum
Picrophilus_torridus
Ferroplasma_acidarmanus
Natronomonas_pharaonis
Haloarcula_marismortui
Halorhabdus_utahensis
Halomicrobium_mukohataei
Natrialba_magadii
Haloterrigena_turkmenica
Halalkalicoccus_jeotgali
Halorubrum_lacusprofundi
Haloquadratum_walsbyi
Haloferax_volcanii
Halogeometricum_borinquense
Halobacterium_sp_NRC1
Candidatus_Nanosalina_sp_J07AB43
Entamoeba_histolytica
Oryza_sativa
Arabidopsis_thaliana
Encephalitozoon_cuniculi
Dictyostelium_discoideum
Trichomonas_vaginalis
Thalassiosira_pseudonana
Phaeodactylum_tricornutum
Trypanosoma_brucei
Leishmania_braziliensis
Giardia_lamblia
Tetrahymena_thermophila
Theileria_parva
Babesia_bovis
Ustilago_maydis
Podospora_anserina
Aspergillus_fumigatus
Saccharomyces_cerevisiae
Candida_albicans
Ashbya_gossypii
Mus_musculus
Bos_taurus
Caenorhabditis_elegans
Brugia_malayi
Drosophila_melanogaster
Apis_mellifera
Methanothermus_fervidus
Methanosphaera_stadtmanae
Methanobrevibacter_smithii_DSM_2375
Methanobrevibacter_smithii_ATCC_35061
Methanobrevibacter_ruminantium
Methanothermobacter_thermautotrophicus
Methanothermobacter_marburgensis
Methanopyrus_kandleri_AV19
Methanocaldococcus_vulcanius
Methanocaldococcus_jannaschii
Methanocaldococcus_fervens
Methanocaldococcus_sp_FS406_22
Methanocaldococcus_infernus
Methanothermococcus_okinawensis
Methanococcus_aeolicus
Methanococcus_voltae
Methanococcus_vannielii
Methanococcus_maripaludis_S2
Methanococcus_maripaludis_C7
Methanococcus_maripaludis_C6
Methanococcus_maripaludis_C5
Methanosaeta_thermophila
Methanocorpusculum_labreanum
Methanoculleus_marisnigri
Methanoplanus_petrolearius
Methanospirillum_hungatei
Methanosphaerula_palustris
Candidatus_Methanoregula_boonei
Methanohalophilus_mahii
Methanohalobium_evestigatum
Methanococcoides_burtonii
Methanosarcina_mazei
Methanosarcina_barkeri
Methanosarcina_acetivorans
Archaeoglobus_profundus
Ferroglobus_placidus
Archaeoglobus_fulgidus
Thermococcus_sibiricus
Thermococcus_barophilus
Pyrococcus_furiosus
Pyrococcus_horikoshii
Pyrococcus_abyssi
Thermococcus_kodakarensis
Thermococcus_onnurineus
Thermococcus_sp_AM4
Thermococcus_gammatolerans
Opitutus_terrae
Coraliomargarita_akajimensis
Verrucomicrobium_spinosum
Chthoniobacter_flavus
Gemmata_obscuriglobus
Planctomyces_limnophilus
Rhodopirellula_baltica
Blastopirellula_marina
Thermus_thermophilus
Rickettsiella_grylli
Marinomonas_sp__MWYL1
Deinococcus_radiodurans
Roseiflexus_castenholzii
Chloroflexus_aggregans
Methylacidiphilum_infernorum
Akkermansia_muciniphila
Dehalogenimonas_lykanthroporepellens
Caldicellulosiruptor_saccharolyticus
Bdellovibrio_bacteriovorus
Treponema_pallidum
Treponema_denticola
Spirochaeta_thermophila
Spirochaeta_smaragdinae
Borrelia_spielmanii
Borrelia_garinii
Chloroherpeton_thalassium
Pelodictyon_phaeoclathratiforme
Prosthecochloris_aestuarii
Chlorobium_phaeobacteroides
Chlorobaculum_parvum
Salinibacter_ruber
Pedobacter_heparinus
Chitinophaga_pinensis
Dyadobacter_fermentans
Cytophaga_hutchinsonii
Gramella_forsetii
Flavobacterium_johnsoniae
Robiginitalea_biformata
Capnocytophaga_ochracea
Porphyromonas_gingivalis
Bacteroides_fragilis
Thermobifida_fusca
Streptomyces_coelicolor
Salinispora_arenicola
Kribbella_flavida
Nocardia_farcinica
Corynebacterium_efficiens
Clavibacter_michiganensis
Kineococcus_radiotolerans
Leifsonia_xyli
Kocuria_rhizophila
Brachybacterium_faecium
Saccharopolyspora_erythraea
Rhodococcus_erythropolis
Mycobacterium_smegmatis
Propionibacterium_acnes
Bifidobacterium_adolescentis
Arthrobacter_aurescens
Acidothermus_cellulolyticus
Acidimicrobium_ferrooxidans
Thermomicrobium_roseum
Sphaerobacter_thermophilus
Leptospira_interrogans
Leptospira_biflexa
Finegoldia_magna
Thermoanaerobacter_pseudethanolicus
Clostridium_acetobutylicum
Staphylococcus_aureus
Macrococcus_caseolyticus
Geobacillus_thermodenitrificans
Anoxybacillus_flavithermus
Alkaliphilus_metalliredigens
Ureaplasma_parvum
Mesoplasma_florum
Candidatus_Phytoplasma_australiense
Acholeplasma_laidlawii
1.0
